# Atrial Pacing Negatively Affects Left Atrial Morphological and Functional Parameters Similarly to Atrioventricular Dyssynchrony

**DOI:** 10.3390/medicina60030503

**Published:** 2024-03-19

**Authors:** Mindaugas Viezelis, Gintare Neverauskaite-Piliponiene, Agne Marcinkeviciene, Tomas Kazakevicius, Vytautas Zabiela, Vilius Kviesulaitis, Renaldas Jurkevicius, Aras Puodziukynas

**Affiliations:** 1 Department of Cardiology, Lithuanian University of Health Sciences, A. Mickeviciaus Str. 7, LT-44307 Kaunas, Lithuania; agne.marcinkeviciene@kaunoklinikos.lt (A.M.); tomas.kazakevicius@lsmuni.lt (T.K.); vytautas.zabiela@lsmuni.lt (V.Z.); renaldas.jurkevicius@lsmuni.lt (R.J.); aras.puodziukynas@lsmuni.lt (A.P.); 2 Department of Cardiology, Hospital of Lithuanian University of Health Sciences Kaunas Clinics, Eiveniu Str. 2, LT-50161 Kaunas, Lithuania; gintare.piliponiene@kaunoklinikos.lt (G.N.-P.); vilius.kviesulaitis@kaunoklinikos.lt (V.K.); 3 Institute of Cardiology, Lithuanian University of Health Sciences, Sukileliu Str. 15, LT-44307 Kaunas, Lithuania

**Keywords:** cardiac pacing, left atrial function, strain

## Abstract

*Background and Objectives*: Atrioventricular (AV) dyssynchrony as well as atrial and ventricular pacing affect left atrial (LA) function. We conducted a study evaluating the effect of atrial and ventricular pacing on LA morphological and functional changes after dual-chamber pacemaker implantation. *Materials and Methods*: The study prospectively enrolled 121 subjects who had a dual-chamber pacemaker implanted due to sinus node disease (SND) or atrioventricular block (AVB). Subjects were divided into three groups based on indication and pacemaker programming: (1) SND DDDR 60; (2) AVB DDD 60 and (3) AVB DDD 40. Subjects were invited to one- and three-month follow-up visits. Three subsets based on pacing burden were analyzed: (1) high atrial (A) low ventricular (V); (2) high A, high V and (3) low A, high V. LA function was assessed from volumetric parameters and measured strains from echocardiography. *Results*: The high A, low V group consisted of 38 subjects; while high A, high V had 26 and low A, high V had 23. A significant decrease in reservoir and contractile LA strain parameters were only observed in the high A, low V pacing group after three months (reservoir 25.9 ± 10.3% vs. 21.1 ± 9.9%, *p* = 0.003, contractile −14.0 ± 9.0% vs. −11.1 ± 7.8, *p* = 0.018). While the re-established atrioventricular synchrony in the low A, high V group maintained reservoir LA strain at the baseline level after three months (21.4 ± 10.4% vs. 22.5 ± 10.4%, *p* = 0.975); in the high A, high V group, a further trend to decrease was noted (20.3 ± 8.9% vs. 18.7 ± 8.3%, *p* = 0.231). *Conclusions*: High atrial pacing burden independently of atrioventricular dyssynchrony and ventricular pacing impairs LA functional and morphological parameters. Changes appear soon after pacemaker implantation and are maintained.

## 1. Introduction

Atrioventricular block (AVB) and sinus node dysfunction (SND) can be effectively treated by cardiac pacing. Dual-chamber cardiac pacemaker implantation remains the first choice in cases of preserved left ventricular (LV) function [1,2]. Though dual-chamber pacing is more physiological by maintaining atrioventricular synchrony [3], it has been shown that right ventricular (RV) pacing has a detrimental effect on ventricular function [4,5]. This increases the risk of heart failure, atrial fibrillation (AF) and death. High RV pacing burden of more than 40% impairs LV function by causing interventricular and intraventricular dyssynchrony [6,7]. Studies have demonstrated that left atrial (LA) function can also be affected by RV pacing due to induced LV dyssynchrony and corresponding LA dyssynchrony or atrioventricular dyssynchrony [8,9]. Atrial pacing by itself can also cause deterioration of LA function. That can result in arrhythmias regardless of reduced or preserved LV systolic function [10,11,12,13,14,15]. In a population of patients with reduced LV ejection fraction (LVEF) receiving cardiac resynchronization therapy, higher right atrial (RA) pacing burden can lead to worsening of LA function, higher risk of AF and HF worsening [13]. Exact interplay between atrioventricular dyssynchrony, atrial and ventricular pacing burden and their impact on LA function have not been assessed to date. By evaluating atrial remodeling in different patient groups, a more optimal pacing strategy could be developed.

We conducted a study evaluating the effect of atrial and ventricular pacing on LA morphological and functional changes after dual-chamber pacemaker implantation.

## 2. Materials and Methods

### 2.1. Study Subjects

The study enrolled subjects from June 2020 to November 2021 at Lithuanian University of Health Sciences, Cardiology Department. A total of 121 subjects were enrolled prospectively. Indications for pacemaker implantation were advanced AVB and significant SND. In every case, a transvenous dual-chamber pacemaker was implanted. Pacemaker leads were positioned primarily targeting the RA appendage and RV septum under fluoroscopy guidance. Subjects were excluded if they had a known history of persistent AF, LV EF < 50%, significant structural heart disease or were not able to continue to follow-up visits. Subjects were divided into three groups based on indication and pacemaker programming: (1) SND DDDR 60; (2) AVB DDD 60 and (3) AVB DDD 40. For the SND indication, a right ventricular pacing avoiding programming options was used, allowing an AV delay of up to 280 ms. For the AVB groups, nominal AV delays of 150 ms for the sensed AV delay and 200 ms for the paced were chosen. Clinical characteristics data regarding age, gender and previous medical history were collected during the enrolment. The subjects were discharged the day after pacemaker implantation. Follow-up visits were performed after one and three months. The Lithuanian National Ethical Committee approved the study. All patients gave their signed informed consent.

### 2.2. Echocardiography

A two-dimensional transthoracic echocardiography (TTE) and tissue Doppler imaging (TDI) were performed. A standard ultrasound system (model EPIQ 7, Philips Medical Systems, Andover, MA, USA) was used. The baseline echocardiography was carried out the day after pacemaker implantation and subsequently during one- and three-month follow-up visits. Investigations were performed by experienced investigators.

High quality 4-, 3- and 2-chamber apical and parasternal views were obtained. Clips of four cardiac beats were recorded. Image acquisition was optimized to maintain high frame rate while avoiding LA foreshortening. Analysis was carried out by one investigator. The reader was blinded to other study data including pacing burden.

### 2.3. Left Atrium Analysis

**Figure 1:**
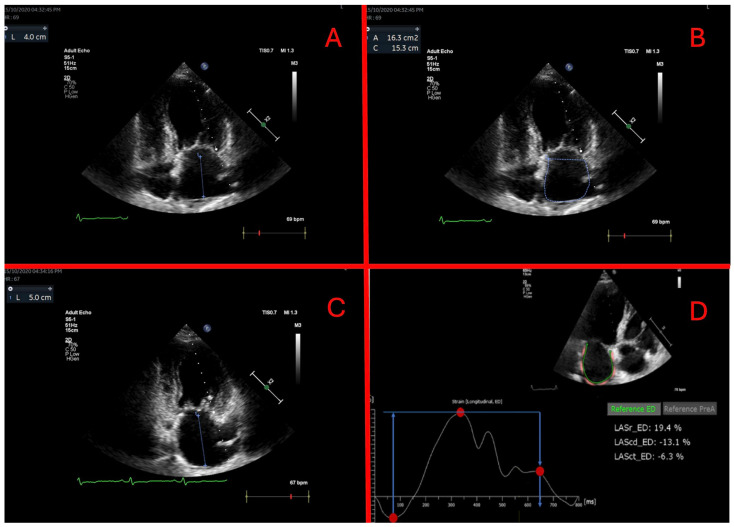
Left atrium morphometric measurements and strain analysis. (**A**) left atrial length in 4-chamber view at end-diastole; (**B**) left atrial area in 4-chamber view at end-diastole; (**C**) left atrial length in 2-chamber view at pre-atrial contraction; (**D**) left atrial strain measurements. Reservoir strain is calculated as a positive value, conduit and contractile strains as negative values.

LA volumetric parameters were obtained by the area–length method (LA volume = (4-chamber area) × (2-chamber area) × 0.85/atrial length) in apical 4- and 2-chamber projections based on recommendations from the American Society of Echocardiography [16]. An atrial appendage and pulmonary veins were excluded from endocardial tracings. LA length was measured as distance from the mitral annulus to the posterior wall. Tracings at an end-systole, just before opening of the mitral valve, were used to calculate the LA maximal volume (LAmax), at the start of a P-wave—the LA pre-atrial contraction volume (LAp)—and at an end-diastole, just after mitral valve closure—the LA minimum volume (LAmin). LA emptying fractions were calculated as follows: (1) passive—(LAmax − LAp)/LAmax × 100%; (2) active—(LAp − LAmin)/LAp × 100% and (3) total—(LAmax − LAmin)/LAmax × 100%. Echocardiographic parameters were analyzed using EchoPAC PC (version 112, GE, Horten, Norway). Figure 1 details LA morphometric measurements.

For strain analysis, a Philips QLAB (version 15.0 Philips Medical Systems, Andover, MA, USA) was used. For the reference, an atrial cycle was chosen [17]. An endocardial border was traced automatically in the 4- and 2-chamber projections. If needed, tracings were manually adjusted. Reservoir strain is calculated as a positive value and conduit and contractile strains as negative values. Their values were calculated as the difference between two measured points. Strain measurement is detailed in Figure 1.

A pulsed-wave Doppler was used to measure E and A waves and their ratio for mitral inflow pattern evaluation. Pulsed-wave tissue Doppler imaging was used to obtain lateral and septal mitral annular velocities (e′), and the values were averaged. The E/e′ ratio was measured and calculated as previously recommended [18]. The stiffness index was evaluated as the ratio of E/e′ to the reservoir strain [19].

### 2.4. Follow-Up Visit Procedure

Subjects attended follow-up visits after one and three months. Echocardiography was performed in the same implanting center. Pacemakers were interrogated noting atrial and ventricular pacing burdens.

A cut-off pacing threshold of 40% based on historical data [5,6] was chosen for atrial and ventricular pacing. To investigate the RA and RV pacing effects and differences for LA functional parameters, we investigated groups with high RA and low RV, low RA and high RV and high RA and high RV pacing burdens.

### 2.5. Statistical Analysis

Continuous variables are presented as mean ± SD and categorical as percentages, unless stated otherwise. The data distribution was determined using the Shapiro–Wilk test. The Mann–Whitney U test or Student’s *t*-test were used to compare continuous variables. Data from follow-up visits were analyzed as paired samples using the Wilcoxon test or Student’s *t*-test as appropriate. The Chi-square test was used to compare categorical variables and the ANOVA model to compare baseline LA parameters between groups. A *p*-value < 0.05 was chosen as the statistical significance threshold. Intraobserver variability for strain parameters was evaluated in 20 random subjects. Identical cine-loops for each view were used, and intraclass correlation was calculated. For statistical analysis, SPSS Statistics 24.0 (IBM Corp, Armonk, NY, USA) was used.

## 3. Results

### 3.1. Pacing Distribution

**Figure 2:**
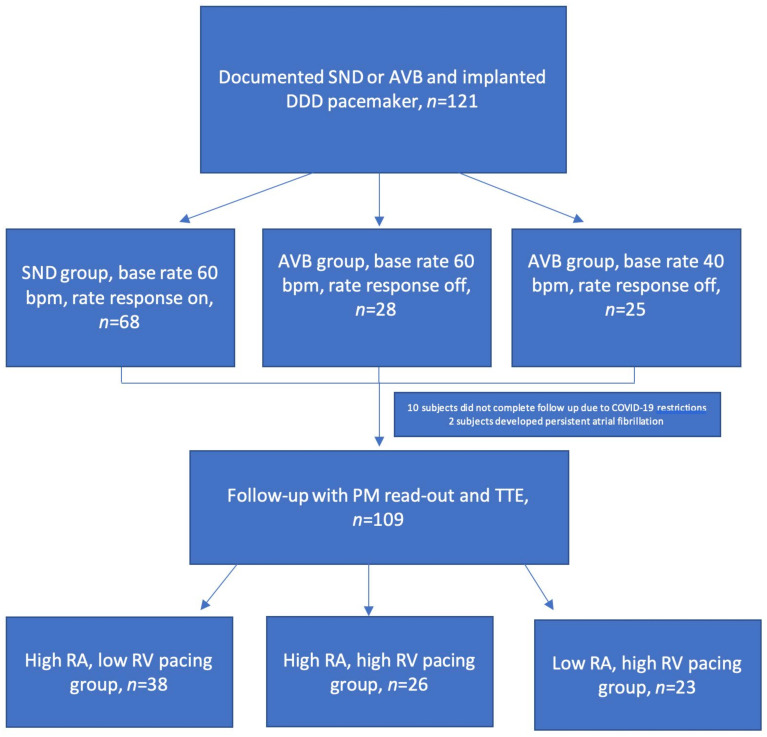
Flowchart of subject distribution. AVB—atrioventricular block; bpm—beats per minute; PM—pacemaker; RA—right atrial, RV—right ventricular; SND—sinus node disease; TTE—transthoracic echocardiography.

In total, 121 subjects (age 74.5 ± 10.4 years; 74 (61.2%) women) were included in the study. There were 68 subjects enrolled with an indication of a SND and 53 with an AVB. The flow of the study subjects is detailed in Figure 2.

**Figure 3:**
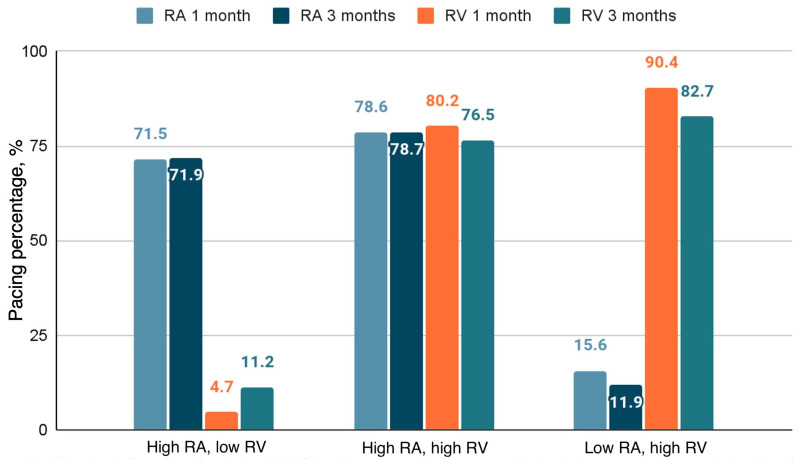
Pacing percentage. High RA, low RV group—atrial pacing > 40%, ventricular pacing < 40%. High RA, high RV group—atrial pacing > 40%, ventricular pacing > 40%. Low RA, high RV group—atrial pacing < 40%, ventricular pacing > 40%. RA—right atrium; RV—right ventricle.

Due to traveling restrictions related to COVID-19, ten subjects did not attend follow-up visits. Persistent atrial fibrillation was detected in two subjects during control visits. They were excluded from further analysis. The remaining 109 subjects fully completed follow up visits with study echocardiography and pacemaker read-out. Atrial and ventricular pacing distributions are shown in Figure 3. The distribution of pacing burden significantly remained unchanged during follow-up period (*p* < 0.001).

Intraobserver variability intraclass correlation coefficients were 0.958 for reservoir, 0.911 for conduit and 0.917 for contractile strains.

### 3.2. Baseline Parameters

The baseline clinical characteristics of the groups analyzed are presented in Table 1. The subjects in the high RA, high RV group were slightly older. There were slightly more males in both high RV pacing groups, and there was a tendency for higher body mass index in the low RA, high RV group. None of the mentioned parameters reached statistical significance. Most subjects had arterial hypertension and were mostly treated with angiotensin-converting enzyme or sartans followed by beta-adrenergic receptor blockers. The paroxysmal AF and coronary artery disease distribution ranged from 30% to 40%. LV size and EF were within the normal range. The mitral inflow pattern showed abnormal relaxation. The distribution of medical history, medications, LV measurements and mitral inflow pattern parameters was not significantly different between groups.

The baseline LA morphological and functional parameters between the analyzed groups are shown in Table 2. A tendency for LAmax and LAmin to be larger in high RV pacing burden groups was noted. However, when indexed to the body surface area, though not significantly, only LAmax in the high RA, high RV group remained larger. Total emptying fractions tended to be lower in high RV pacing groups while the active fraction remained higher in the high RA and low RV group. All strain parameters were lower in high RV pacing groups, though none reached significance. Likewise, the stiffness index was also higher with high RV pacing burden. When comparing the two groups with high RV pacing, a slightly lower value of the stiffness index in a low RA pacing group was noted.

### 3.3. Follow-Up

Volumes tended to increase in all three groups. However, a greater increase was noted in high RV pacing groups (Table 3, Table 4 and Table 5). The most significant change was noted in high RA and high RV pacing groups being reproduced in absolute and indexed values. A trend towards a decrease in all functional LA parameters was noticed in high RA, low RV group, while that in the high RA and high RV group remained similar (Table 4), and that in the low RA, high RV tended to improve. Similarly, a pronounced statistical trend for strain values to decrease was noted in the high RA, low RV group. While lower, a decline in strain values was noted in high RA and high RV group, and a slight trend towards improvement was noticed in the low RA, high RV group. Though the stiffness index decreased in every group, it only reached significance in the high RA and low RV group. Though the same trend of increased E/A ratio was observed in all groups, statistical significance was reached in high RA pacing groups. E/e′ ratio did not show a significant change in any of the groups.

## 4. Discussion

This study investigated how atrial and ventricular pacing based on their burden affects LA function in subjects with preserved LV EF after dual-chamber pacemaker implantation. It provides data regarding impact of atrioventricular dyssynchrony, atrioventricular resynchronization and atrial pacing burden on LA function. As the DDD(R) pacing mode remains the most common method of pacing, it is important to recognize that atrial pacing independently reduces LA function.

It has been previously shown that patients with atrioventricular block develop atrial enlargement. This effect could be related to atrioventricular dyssynchrony [20]. Permanent loss of AV synchrony induced by VVI pacing is associated with mechanical remodeling of the left atrium, which may reverse after the reestablishment of AV synchrony with DDD pacing [21,22,23]. In our study, though not significantly, we have observed greater LA volumes at the baseline in groups that resulted in high RV pacing burden, thus indicating a significant previous AV dyssynchrony. Worse strain parameters representing all three atrial functions were also noted in high RV pacing groups.

LA volume is a known predictor of new HF development [24]. In our study, all LA volumes tended to increase, with the most significant changes appearing in the setting of higher RV pacing burden. The changes were noted as early as the one-month follow-up visit and were maintained during the study. Recently published data from the Danpace II trial [25] have supported a higher base rate of atrial pacing based on clinical expression of symptoms; however, the influence of atrial pacing on structural and functional changes in atria, which can have a late impact on progression of disease, were not evaluated.

It is well established that RV pacing negatively impacts LV function [4,5,6,7,26]. It has been shown that it can also have a detrimental effect on LA function [6,27]. Acute RV apical pacing results in LV dyssynchrony and higher LV filling pressure. Consequently, transmitral inflow is affected during the late LV diastole, which causes a larger volume build-up before the LA systole. Increased LA pressure might reduce longitudinal deformation during ventricular ejection. The atrial shortening during ventricular early filling is also reduced [14]. In our study, though showing trend for improvement after atrioventricular resynchronization in low RA, high RV pacing group, strain parameters did not reach baseline levels of high RA, low RV group at three-month follow-up. In the high RA, high RV group, even after atrioventricular resynchronization, further decrease in strain values were noted. A similar trend was observed with emptying fraction parameters. While low RA, high RV group showed trend for improvement, high RA, high RV remained similar, high RA and low RV group had a tendency for emptying fractions to decrease. That indicates that a positive effect of atrioventricular resynchronization is reduced by RA pacing.

Liang et al. has shown that atrial pacing is related to intra-atrial dyssynchrony. That might limit LA preload contribution to LV stroke volume [27]. Martens et al. investigated an atrial pacing effect on interatrial dyssynchrony in patients undergoing CRT implantation. They noticed a link between higher atrial pacing burden and reduced LA reverse remodeling [13]. We have previously demonstrated that more atrial pacing was linked to negative LA remodeling and worsened systolic and diastolic LA functions in patients with preserved LV EF receiving dual-chamber pacemakers [15]. Though the degree to which different pacing modes, interatrial and AV dyssynchrony, RA pacing burden affected LA function remained unclear.

In our study a decrease in all three strain parameters and increase in volumes were noticed in low right atrial pacing group at the first follow-up and downward trend persisted after three months. These findings could be explained by the introduction of less synchronous atrial contraction and less septal compliance. Furthermore, the interatrial delay, if significant enough, could lead the LA systole against the closed mitral valve. This could additionally lead to increased LA pressure and alter function. It is worth mentioning that RV pacing was not negligible in the low RA, low RV group and could have also influenced LA function.

Both high RV pacing groups had markedly lower strain parameters at the baseline when compared to the low RV pacing group. This suggests that atrioventricular dyssynchrony is a potent factor reducing LA function. However, at follow-up visits, the trends diverged. In the group that maintained low RA pacing, a trend of strain improvement was observed. Though not statistically significant, the numerical increase was noted in all three strains. These observations remained unchanged at the first and second follow-up visits. When analyzing the high RA, high RV pacing group, a trend to decrease regarding all three strain parameters was noticed during follow-up. It appears that re-establishing AV synchrony partly restores LA function by eliminating cannon waves, improving filling and compliance. However, the negative impact of RV pacing on LA function by creating ventricular dyssynchrony [6,14,27,28,29,30] leads to an increase in LA volume and prevents further strain improvement. However, in the setting of both atrial and ventricular pacing, the AV resynchronization benefit on strain parameters appears to be further reduced by interatrial and interventricular dyssynchrony. In addition, standard AV delays could be too short to compensate for introduced interatrial delay by atrial pacing, thus leading to premature valve closure and interruption of transmitral flow [31]. This mechanism could be supported by the observation of greater volume changes in high RV pacing groups, Lower volumes were observed in low RV pacing group where intrinsic conduction dominated. However, our study was not designed to evaluate changes in the setting of different AV delays with high RV pacing burden.

A trend for the stiffness index to increase was noted in all the groups. Though dependent on mitral inflow and mitral annular velocities, the trend was mostly driven by the decrease in reservoir strain value that was most prominent in the high RA, low RV pacing group. A recent study linked an increased LA stiffness index with higher risk for hospitalization for heart failure and all-cause mortality during a median 6-year follow-up. The prognostic role was even more pronounced than that of left ventricular filling pressure indexes [32].

We believe our findings could be important in clinical practice. To date, besides facilitating intrinsic conduction when available due to well-established knowledge of detrimental effects of RV pacing on LV function [7], no further clear guidelines are available for dual-chamber pacemaker programming [1,33]. The pacing rate is a modifiable factor and should be individualized to the patients’ needs. However, it has been shown that the usually nominal base rate of 60 beats per minute is not modified at implantation despite the indication [34]. Our findings support promoting atrial sensing by programming a minimal acceptable base rate when feasible. When high atrial pacing burden cannot be avoided, an alternative site of pacing could be considered to reduce intra- and interatrial dyssynchrony [13,26,27,35,36,37,38]. Considering AV timing optimization not just to avoid RV pacing but also to correct for interatrial dyssynchrony might further help to preserve LA function [12,16,31]. Avoiding apical RV pacing during implantation [25] and reducing LV dyssynchrony could also help to maintain LV function [28,29]. Conduction system pacing, aiming to benefit from the heart’s intrinsic conduction system, can possibly maintain interventricular synchrony and LV filling pressure. This can avoid increased pressure and LA function decline [39].

### Study Limitations

First, we recognize the modest number of subjects included in the study. Second, a good image quality is required for strain analysis. Third, pacemaker programming was strictly controlled regarding base rate and rate response parameters; however, AV delay was not. Fourth, a relatively short follow-up period prevents long-term conclusions on LA function and arrhythmia risk from being drawn. Fifth, in every group, even if considered low (either RA or RV), pacing burden was not negligible. Sixth, though septal RV pacing was aimed for during implantation, imprecise lead placement could have influenced atrial and ventricular function.

## 5. Conclusions

High RA pacing burden independently of atrioventricular dyssynchrony and RV pacing impairs LA functional and morphological parameters. Changes appear soon after pacemaker implantation and are maintained during the follow-up.

## Figures and Tables

**Table 1:** Baseline parameters.

	High A Low V ^a^ (*n* = 38)	High A High V ^b^ (*n* = 26)	Low A High V ^c^ (*n* = 23)	*p*
Age, years	73.6 ± 10.2	78.7 ± 8.3	73.7 ± 9.8	0.104
Male, *n* (%)	12 (31.7)	11 (42.3)	9 (39.1)	0.658
BSA, m^2^	1.89 ± 0.2	1.91 ± 0.22	1.98 ± 0.23	0.472
BMI, kg/m^2^	28.4 ± 4.2	29.0 ± 5.8	30.3 ± 5.8	0.494
Medical history				
Hypertension, *n* (%)	35 (92.1)	24 (92.3)	21 (91.3)	0.991
Diabetes mellitus, *n* (%)	3 (7.9)	5 (19.2)	4 (17.4)	0.366
PAF, *n* (%)	15 (39.4)	10 (38.5)	8 (34.8)	0.933
CAD, *n* (%)	14 (36.8)	8 (30.8)	8 (34.8)	0.881
Chronic renal failure, *n* (%)	2 (5.2)	3 (11.5)	1 (4.3)	0.532
Medications				
ACE inhibitors/ARB, *n* (%)	36 (94.7)	24 (92.3)	21 (91.3)	0.861
BAB, *n* (%)	29 (76.3)	20 (76.9)	18 (78.2)	0.985
MRA, *n* (%)	10 (26.3)	6 (23.1)	6 (26.1)	0.953
non-MRA diuretic, *n* (%)	17 (44.7)	13 (50.0)	10 (43.4)	0.882
Non-dihydropyridine CCB, *n* (%)	1 (2.6)	0 (0)	0 (0)	0.521
Statin, *n* (%)	12 (31.6)	10 (38.5)	9 (39.1)	0.784
LV parameters				
LVEDD, mm	49.2 ± 4.5	49.8 ± 5.1	51.2 ± 5.7	0.360
LVEDD index, mL/m^2^	26.2 ± 2.2	26.4 ± 2.6	25.6 ± 2.9	0.867
LV EF, %	58.4 ± 4.9	58.9 ± 5.0	57.6 ± 5.2	0.768
E/A	0.89 ± 0.37	0.84 ± 0.43	0.85 ± 0.41	0.856
E/e′	8.4 ± 2.6	10.4 ± 4.5	9.8 ± 4.0	0.547

ACE—angiotensin-converting enzyme; ARB—angiotensin receptor blockers; BAB—beta-adrenergic receptor blockers; BMI—body mass index; BSA—body surface area; CAD—coronary artery disease; CCB—calcium channel blocker; EF—ejection fraction; LV—left ventricular, LVEDD—left ventricle end-diastolic diameter; MRA—mineralocorticoid receptor antagonists, PAF—paroxysmal atrial fibrillation. ^a^ High A, low V group—atrial pacing > 40%, ventricular pacing < 40%. ^b^ High A, high V group—atrial pacing > 40%, ventricular pacing > 40%. ^c^ Low A, high V group—atrial pacing < 40%, ventricular pacing > 40%.

**Table 2:** Baseline left atrium parameters.

	High A Low V ^a^ (*n* = 38)	High A High V ^b^ (*n* = 26)	Low A High V ^c^ (*n* = 23)	*p*
Volumes				
LAmax, mL	73.2 ± 17.3	77.8 ± 24.2	76.1 ± 26.1	0.684
LAmax index, mL/m^2^	38.7 ± 7.9	40.6 ± 10.8	37.8 ± 10.5	0.558
LAp, mL	53.7 ± 14.3	53.4 ± 19.3	55.1 ± 18.5	0.939
LAp index, mL/m^2^	28.3 ± 6.8	27.8 ± 8.9	27.5 ± 7.6	0.914
LAmin, mL	38.2 ± 11.8	40.3 ± 16.7	42.2 ± 15.1	0.592
LAmin index, mL/m^2^	20.2 ± 5.9	20.9 ± 7.7	21.0 ± 6.4	0.861
Emptying fractions				
Total, %	48.1 ± 8.2	47.3 ± 9.0	44.4 ± 7.8	0.133
Passive, %	26.7 ± 8.2	28.8 ± 9.1	27.2 ± 8.4	0.634
Active, %	29.2 ± 8.7	25.8 ± 10.4	23.7 ± 5.8	0.450
Strains				
Reservoir, %	25.9 ± 10.3	20.3 ± 8.9	21.4 ± 10.4	0.054
Conduit, %	−11.9 ± 5.3	−8.8 ± 4.3	−10.4 ± 8.1	0.090
Contractile, %	−14.0 ± 9.0	−11.4 ± 8.5	−10.9 ± 8.6	0.345
Stiffness index	0.41 ± 0.27	0.72 ± 0.67	0.62 ± 0.58	0.040

LAmax—maximal left atrium volume; LAmin—minimal left atrium volume, LAp—pre-atrial contraction left atrium volume; A—atrium, V—ventricle. ^a^ High A, low V group—atrial pacing > 40%, ventricular pacing < 40%. ^b^ High A, high V group—atrial pacing > 40%, ventricular pacing > 40%. ^c^ Low A, high V group—atrial pacing < 40%, ventricular pacing > 40%.

**Table 3:** High atrial and low ventricular pacing group.

	Baseline	1 Month	3 Months	*p* Baseline vs. 1 Month	*p* Baseline vs. 3 Months
Volumes					
LAmax, mL	73.2 ± 17.3	77.8 ± 21.1	75.8 ± 20.1	0.442	0.367
LAmax index, mL/m^2^	38.7 ± 7.9	41.0 ± 10.4	40.1 ± 10.0	0.424	0.376
LAp, mL	53.7 ± 14.3	55.5 ± 16.2	57.3 ± 17.5	0.294	0.161
LAp index, mL/m^2^	28.3 ± 6.8	29.3 ± 8.2	30.2 ± 8.4	0.261	0.186
LAmin, mL	38.2 ± 11.8	41.3 ± 14.6	42.7 ± 13.7	0.169	0.038
LAmin index, mL/m^2^	20.2 ± 5.9	21.7 ± 7.3	22.6 ± 7.5	0.190	0.039
Emptying fractions					
Total, %	48.1 ± 8.2	47.6 ± 8.6	44.9 ± 9.8	0.678	0.033
Passive, %	26.7 ± 8.2	28.6 ± 9.4	24.5 ± 9.7	0.398	0.401
Active, %	29.2 ± 8.7	26.5 ± 8.5	25.7 ± 8.9	0.076	0.043
Strains					
Reservoir, %	25.9 ± 10.3	24.4 ± 9.5	21.1 ± 9.9	0.315	0.003
Conduit, %	−11.9 ± 5.3	−11.8 ± 6.4	−10.0 ± 5.3	0.798	0.086
Contractile, %	−14.0 ± 9.0	−12.7 ± 7.0	−11.1 ± 7.8	0.342	0.018
Stiffness index	0.41 ± 0.27	0.46 ± 0.33	0.67 ± 0.65	0.478	0.001
Mitral inflow					
E/A	0.89 ± 0.37	1.02 ± 0.44	0.99 ± 0.43	0.008	0.043
E/e′	8.4 ± 2.6	9.2 ± 4.5	9.0 ± 4.0	0.598	0.914

LAmax—maximal left atrium volume, LAmin—minimal left atrium volume, LAp—pre-atrial contraction left atrium volume.

**Table 4:** High atrial high ventricular pacing group.

	Baseline	1 Month	3 Months	*p* Baseline vs. 1 Month	*p* Baseline vs. 3 Months
Volumes					
LAmax, mL	77.8 ± 24.2	85.5 ± 24.1	87.4 ± 19.6	0.012	0.085
LAmax index, mL/m^2^	40.6 ± 10.8	44.5 ± 10.3	45.4 ± 10.2	0.015	0.073
LAp, mL	53.4 ± 19.3	61.6 ± 18.2	62.5 ± 19.6	0.001	0.086
LAp index, mL/m^2^	27.8 ± 8.9	32.1 ± 8.1	32.1 ± 8.4	0.001	0.052
LAmin, mL	40.3 ± 16.7	43.6 ± 15.9	45.8 ± 15.8	0.047	0.224
LAmin index, mL/m^2^	20.9 ± 7.7	22.7 ± 7.5	23.3 ± 8.0	0.032	0.224
Emptying fractions					
Total, %	47.3 ± 9.0	49.8 ± 8.7	48.3 ± 9.5	0.482	0.964
Passive, %	28.8 ± 9.1	27.9 ± 8.4	28.6 ± 9.7	0.634	0.914
Active, %	25.8 ± 10.4	27.4 ± 9.4	27.6 ± 7.8	0.631	0.573
Strains					
Reservoir, %	20.3 ± 8.9	18.9 ± 7.9	18.7 ± 8.3	0.112	0.231
Conduit, %	−8.8 ± 4.3	−8.5 ± 4.6	−8.4 ± 5.3	0.513	0.738
Contractile, %	−11.4 ± 8.5	−10.3 ± 7.8	−10.0 ± 7.5	0.385	0.988
Stiffness index	0.72 ± 0.67	0.79 ± 0.63	0.90 ± 0.89	0.096	0.340
Mitral inflow					
E/A	0.84 ± 0.43	1.02 ± 0.41	1.05 ± 0.46	0.021	0.365
E/e′	10.4 ± 4.5	10.6 ± 4.8	11.5 ± 4.7	0.864	0.512

LAmax—maximal left atrium volume, LAmin—minimal left atrium volume, LAp—pre-atrial contraction left atrium volume.

**Table 5:** Low atrial high ventricular pacing group.

	Baseline	1 Month	3 Months	*p* Baseline vs. 1 Month	*p* Baseline vs. 3 Months
Volumes					
LAmax, mL	76.1 ± 26.1	85.1 ± 25.3	88.1 ± 28.3	0.022	0.030
LAmax index, mL/m^2^	37.8 ± 10.5	45.0 ± 11.1	43.2 ± 11.7	0.016	0.027
LAp, mL	55.1 ± 18.5	61.9 ± 15.9	62.1 ± 20.8	0.117	0.397
LAp index, mL/m^2^	27.5 ± 7.6	31.1 ± 7.5	30.6 ± 8.6	0.091	0.433
LAmin, mL	42.2 ± 15.1	46.2 ± 13.8	45.0 ± 19.0	0.268	0.925
LAmin index, mL/m^2^	21.0 ± 6.4	23.2 ± 6.8	22.1 ± 7.9	0.251	0.875
Emptying fractions					
Total, %	44.4 ± 7.8	48.3 ± 10.1	49.0 ± 6.1	0.117	0.064
Passive, %	27.2 ± 8.4	30.3 ± 9.8	28.5 ± 7.3	0.136	0.778
Active, %	23.7 ± 5.8	25.7 ± 10.3	28.1 ± 9.7	0.573	0.331
Strains					
Reservoir, %	21.4 ± 10.4	24.3 ± 11.8	22.5 ± 10.4	0.287	0.975
Conduit strain, %	−10.4 ± 8.1	−11.3 ± 6.8	−11.0 ± 6.6	0.124	0.638
Contractile strain, %	−10.9 ± 8.6	−13.0 ± 10.1	−11.5 ± 7.1	0.653	0.683
Stiffness index	0.62 ± 0.58	0.56 ± 0.25	0.73 ± 0.54	0.776	0.937
Mitral inflow					
E/A	0.85 ± 0.41	0.92 ± 0.43	0.98 ± 0.47	0.861	0.287
E/e′	9.8 ± 4.0	10.2 ± 4.5	10.6 ± 4.2	0.798	0.913

LAmax—maximal left atrium volume, LAmin—minimal left atrium volume, LAp—pre-atrial contraction left atrium volume.

## Data Availability

Data are contained within the article.
